# A questionnaire survey of healthcare access and dietary habits in a rural Japanese community: implications for potential community pharmacy roles

**DOI:** 10.1186/s40780-025-00524-4

**Published:** 2025-12-13

**Authors:** Hayato Kizaki, Maho Tsukamoto, Mari Yamada, Sana Ito, Megumi Sasaki, Kentaro Sakamoto, Yuki Ikeda, Koji Miyamoto, Haru Iino, Satoko Hori

**Affiliations:** 1 Division of Drug Informatics, Keio University Faculty of Pharmacy, 1-5-30 Shibakoen, Minato-ku, Tokyo, 105-8512 Japan; 2 Kyowa Chemical Co., Ltd, 2-26-2 Izumi, Higashi-ku, Nagoya, Aichi 461-0001 Japan; 3 Toyone Village Office, 2 Warabidaira, Shimokurokawa, Toyone, Aichi 449-0403 Japan

**Keywords:** Rural health, Healthcare disparities, Food accessibility, Mountainous area, Nutritional status, Cross-sectional studies, Dental health surveys

## Abstract

**Background:**

Residents in rural mountainous areas face significant challenges in accessing healthcare and food resources, affecting their dietary intake, nutritional status, and overall health. However, detailed evidence on the interplay between access limitations, dietary habits, and nutritional status is limited, hindering the development of effective, equity-focused interventions. This study aimed to evaluate healthcare access, food procurement practices, and dietary intake in such a community to identify key challenges and opportunities for sustainable health support.

**Methods:**

A cross-sectional survey was conducted targeting residents aged ≥ 20 years in a rural Japanese village. Two questionnaires were administered: a food frequency questionnaire based on food groups (FFQg) and a questionnaire we developed that assessed healthcare utilization and food procurement. Macronutrient balance was considered optimal when the intake ratios of the three macronutrients were within the recommended ranges (proteins: 13–20%, fats: 20–30%, carbohydrates: 50–65%).

**Results:**

Of 115 respondents (58 males, 57 females), 51.3% shopped for food weekly, with 65.5% requiring 1–2 h of one-way travel time to reach stores. While 86.0% had annual health checkups, only 56.1% received regular dental checkups. Only 25.2% achieved an optimal macronutrient balance for all three macronutrients. Among respondents with hypertension (28.3%), the mean salt intake was 11.3 g, which was higher than that of non-hypertensive residents (*p* < 0.05). A significant correlation was observed between poor chewing ability and lower subjective health status (*p* < 0.05).

**Conclusions:**

Residents in this rural mountainous community face significant challenges in healthcare and food access, which are linked to suboptimal dietary intake and health issues. These findings highlight the need to develop community-based health-support systems that can provide targeted nutritional support and health counseling. In the Japanese context, the health-support functions of community pharmacies represent one potential resource that could be leveraged for this purpose, but further research is needed to determine how such services can be effectively implemented to help mitigate health disparities in underserved populations.

## Introduction

Rural and mountainous areas, often characterized by significant depopulation, pose unique challenges regarding healthcare access and food availability, contributing to health disparities compared to urban populations [1–5]. The combined effects of geographical isolation and declining population density in these regions are further compounded by the aging population demographic shifts observed in many countries. In Japan, where an aging population coincides with geographically dispersed settlements, particularly in mountainous regions, access to healthcare and nutritious food becomes increasingly crucial for health equity. The projected 31% increase in the number of depopulated areas in Japan between 2020 and 2050 [6] further exacerbates these challenges, particularly for older residents facing mobility limitations and reduced access to transportation.

Some research has indicated a correlation between distance to the nearest food store and decreased dietary variety among older adults in rural Japan [7]. Reliance on infrequent, large-volume grocery shopping trips may lead to greater consumption of processed and shelf-stable foods, potentially contributing to dietary imbalances. Limited access to fresh and nutritious food is a longstanding concern [8], as it may significantly impact the dietary intake, nutritional status, and overall health of residents.

In addition to food access challenges, limited access to healthcare services poses a significant barrier to maintaining good health in rural mountainous regions [9, 10]. The scarcity of medical professionals and facilities, coupled with extended travel times to reach them, can lead to delayed diagnoses, inadequate management of chronic conditions, and reduced utilization of preventative care.

However, while studies have examined the relationship between food access and dietary intake [7, 11, 12], a comprehensive understanding of the interplay between healthcare access, including access to pharmaceutical services, food availability, dietary habits, and nutritional outcomes, particularly in these communities remains limited. This knowledge gap hinders the development of effective health interventions, particularly those that could leverage the expanding role of community pharmacies in Japan. Nationwide, there is a growing expectation for pharmacies to evolve into health-support hubs, offering a promising, accessible resource for addressing public health needs. Therefore, a comprehensive investigation is needed to characterize these local challenges and identify evidence-based opportunities for health interventions, including the potential utilization of community pharmacy.

This study aimed to characterize the challenges in healthcare access, food procurement, and dietary intake in a rural mountainous community to identify evidence-based opportunities for targeted interventions that promote health equity, with a focus on exploring the potential roles of community pharmacies.

## Methods

### Study design and participants

This study used an anonymous, self-administered questionnaire survey targeting all residents aged 20 years or older in Toyone Village, Aichi Prefecture. As of April 2022, Toyone Village had a population of 1,002, with only one internal medicine clinic, one dental clinic, and no pharmacies. All residents were invited to participate through direct outreach at the Toyone Village Health Center and by distributing flyers to all households. Those who read the explanatory documents and consented to participate completed the questionnaire.

### Questionnaire survey

The survey consisted of a food frequency questionnaire based on food groups (FFQg) and an additional questionnaire we developed that assessed dietary habits and healthcare resource utilization. The FFQg was developed by Takahashi et al. for the Japanese population [13] and assessed typical weekly dietary and physical activity patterns. This FFQg has been validated for estimating intakes of several food groups and nutrients in Japanese adults, although its specific validity in older, rural populations with potentially unique dietary habits warrants careful consideration when interpreting results.

The self-developed questionnaire was developed through extensive discussions between three researchers (Kizaki H., Iino H., Hori S.) and incorporated feedback from a registered dietitian (Tsukamoto M.). A preliminary survey was conducted with a small group of Toyone Village residents to validate the questionnaire for clarity and relevance. To ensure accurate responses in the main survey, assistance was provided by registered dietitians, pharmacists working at community pharmacies, and pharmacy students for participants who had difficulty completing the questionnaire independently.

This questionnaire, written in Japanese, consisted of multiple-choice questions covering four main categories: subjective health status, food procurement, dietary attitudes, and counseling needs. Regarding subjective health status, this was rated on a five-point scale (from “good” to “poor”), and participants reported on their dental health, regular medical and dental visits, and medication use. Food procurement practices were assessed through questions on shopping frequency, store location, travel time to nearest store, mode of transportation, and reliance on homegrown produce. “Attitudes toward meals” included questions regarding meal enjoyment, perceived dietary challenges, and primary meal preparers (self/spouse/children/parents/grandparents/others). Finally, participants were asked about their interest in receiving dietary and nutritional counseling from registered dietitians.

### Statistical analysis

Responses to the self-developed questionnaire were aggregated. Chewing ability was evaluated based on responses to multiple-choice questions regarding dental health status in the self-developed questionnaire. Respondents who reported being able to “chew anything” were placed in the “maintained chewing ability” group, while others were placed in the “decreased chewing ability” group. For subjective health status analysis, the proportion of respondents who rated their health as either “good” or “fairly good” was compared between the chewing ability groups using the chi-square test. The proportion of respondents who underwent regular dental checkups was compared between the groups using the chi-square test.

Responses to the FFQg were analyzed using Excel Eiyou-kun Ver.9 (Kenpakusha), a specialized software, to calculate the participants’ energy and nutrient intakes as well as their physical activity levels. Using these calculated values, vegetable intake (green, yellow, and other vegetables) and salt intake were compared between males and females. Additionally, vegetable intake was compared between the participants who were actively involved in preparing their meals and those who were not. The contribution of the three main macronutrients—carbohydrates, proteins, and fats—to total energy intake was evaluated as percentages, collectively referred to as the “macronutrient distribution.” This distribution was then compared with the target ranges recommended in the Japanese Dietary Reference Intakes: carbohydrates (50–65%), proteins (with lower limits of 13–15% depending on age and sex and an upper limit of 20%), and fats (20–30%). Based on this comparison, participants were categorized as having a “lower than target amount,” “within target,” or “higher than target amount” energy ratio for each macronutrient. Additionally, we analyzed salt intake stratified by hypertension status to examine potential associations between dietary habits and chronic conditions.

### Ethical approval

All the procedures were performed in accordance with the principles of the Declaration of Helsinki. This study was approved by the Research Ethics Review Committee of Keio University Faculty of Pharmacy (accession number 201218-4, 211213-1). Informed consent was obtained from all the participants who completed the questionnaire. The questionnaires were completed anonymously.

## Results

### Basic characteristics of respondents

Responses were obtained from 115 residents (58 males and 57 females), representing approximately 10% of the total population of Toyone Village. People aged 65 and older comprised 38.3% (*n* = 44) of the respondents. The characteristics of the respondents are summarized in Table 1. The respondents’ age distribution was broad. A notable proportion (49.6%, 57 individuals) reported a high level of physical activity, which represents the ratio of the total daily energy expenditure to the basal metabolic rate, as estimated from self-reported activities via the FFQg.

**Table 1:** Basic characteristics of respondents

	Number (%)	
Sex		
Male	58	(50.4)
Female	57	(49.6)
Age		
20s	13	(11.3)
30s	6	(5.2)
40s	17	(14.8)
50s	23	(20.0)
60s	22	(19.1)
70s	30	(26.1)
80s	4	(3.5)
BMI*1		
Underweight	8	(7.0)
Standard	72	(62.6)
Overweight	35	(30.4)
Physical Activity Level*2		
Level I(Low)	25	(21.7)
Level II(Standard)	33	(28.7)
Level III(High)	57	(49.6)

*1 BMI: The Body Mass Index (BMI) is calculated by dividing body weight (kg) by the square of the height (m). Underweight: BMI < 18.5 / Standard: 18.5 ≤ BMI < 25.0 / Overweight: BMI ≥ 25.0

*2 Physical Activity Level: Physical activity level is defined as the ratio of total daily energy expenditure to basal metabolic rate. It is categorized into three levels based on the intensity of the daily activities. **Level I (Low)**: Characterized by a predominantly sedentary lifestyle with mostly static or low-energy activities. **Level II (Standard)**: Involves mainly sedentary work but includes some movement throughout the day, such as standing tasks, customer service, commuting, shopping, housework, and participation in light sports. **Level III (High)**: This applies to individuals whose jobs require frequent movement or standing or those who maintain regular, vigorous exercise habits during their leisure time, such as participating in sports or other physically demanding activities

### Food shopping and dietary habits

**Fig. 1:**
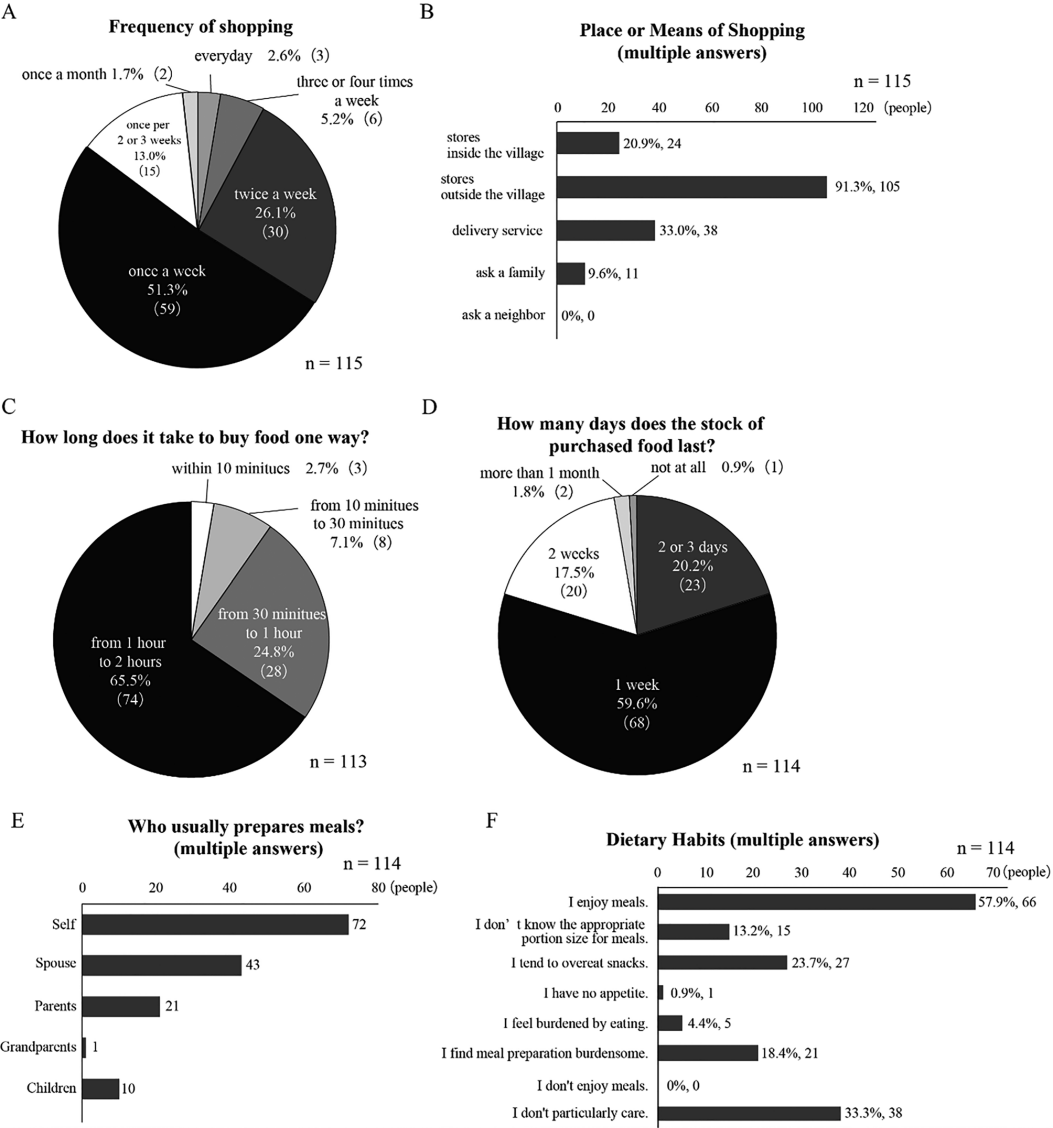
Shopping and food preparation habits among rural residents in Toyone Village. (**A**) Frequency of food shopping among survey respondents (*n* = 115). (**B**) Places or means used for food procurement (multiple answers allowed, *n* = 115). (**C**) One-way travel time required to reach food shopping locations (*n* = 113). (**D**) Duration of household food stock after purchasing (*n* = 114). (**E**) Family members responsible for meal preparation within households (multiple answers allowed, *n* = 114). (**F**) Self-reported dietary habits and attitudes toward eating (multiple answers allowed, *n* = 114)

The most common frequency of food shopping was once a week, as reported by 51.3% (*n* = 59) of the residents (Fig. 1A). A significant majority (91.3%) shopped in stores outside the village (Fig. 1B). Limited store accessibility was evident, as 65.5% of residents reported needing over an hour each way to reach these stores (Fig. 1C). To compensate, 78.9% bought enough food to last more than a week (Fig. 1D). Homegrown produce was common, with a significant number of participants (*n* = 66) growing mainly vegetables. Additionally, 16 residents cultivated rice and fruit, while two engaged in raising chickens or fishing.

Regarding meal preparation, the most common response was that respondents prepared their own meals (Fig. 1E). When analyzed by sex, 37.9% of males prepared their own meals, compared to 89.3% of females. Regarding dietary habits, 57.9% (*n* = 66) of the residents reported enjoying their meals. However, a few residents faced challenges in managing their diets, such as a tendency to overeat snacks (Fig. 1F). Regarding the desire to receive nutritional counseling from registered dietitians, 36.5% of the participants expressed interest.

### Health status and healthcare utilization

**Fig. 2:**
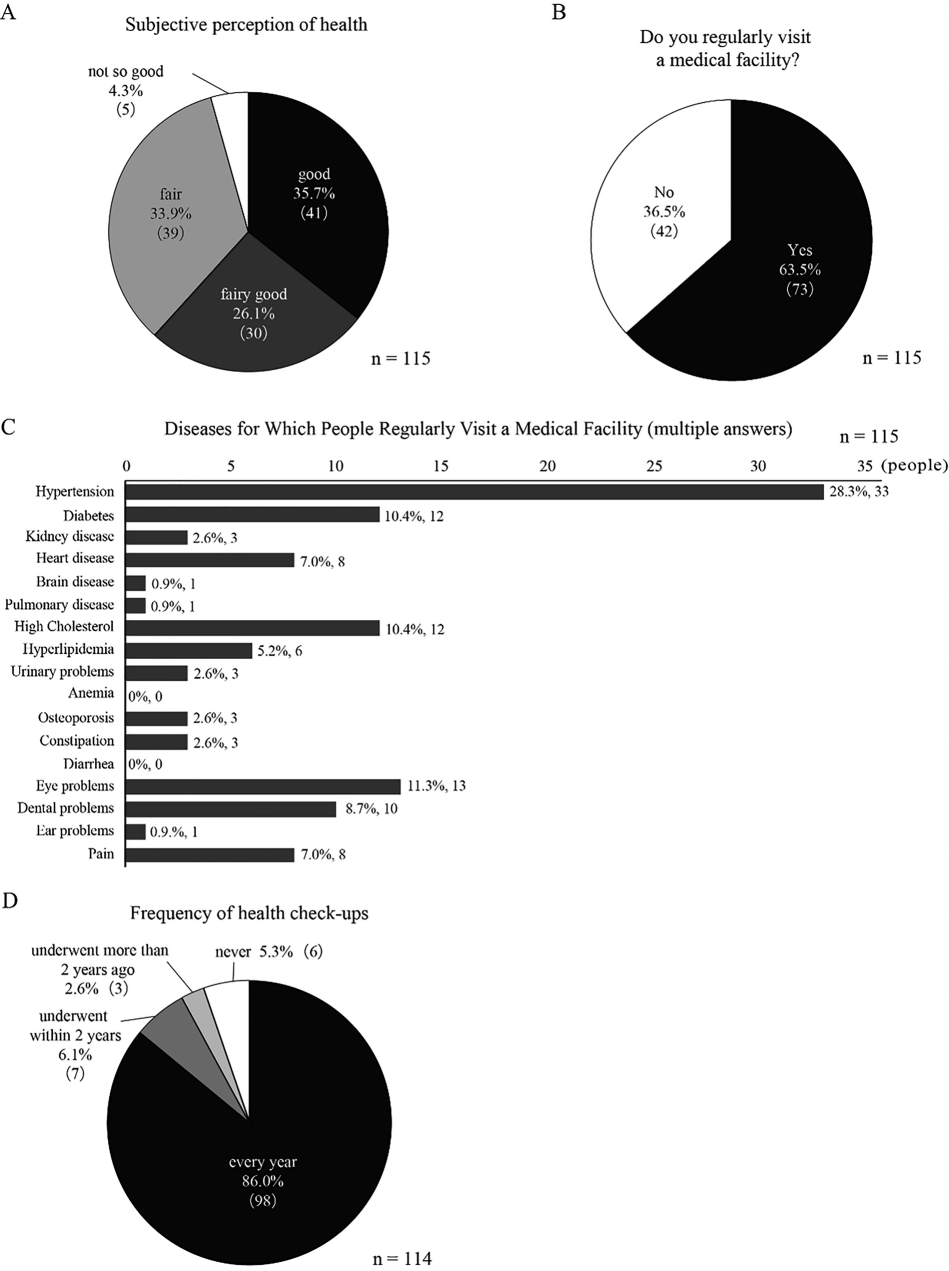
Health status and healthcare access among Toyone Village residents. (**A**) Self-reported subjective perception of health status (*n* = 115). (**B**) Regular medical facility visitation status (*n* = 115). (**C**) Medical conditions for which respondents regularly visit healthcare facilities (multiple answers allowed, *n* = 115). (**D**) Frequency of health check-up participation among respondents (*n* = 114)

Most respondents rated their health as “good,” “fairly good,” or “fair,” with only 4.3% (*n* = 5) reporting that their health was “not so good” (Fig. 2A). A total of 63.5% (*n* = 73) of the participants visited medical facilities regularly (Fig. 2B), with the highest proportion of visits being for hypertension, which affected 28.3% (*n* = 33) of the participants (Fig. 2C). This was followed by dyslipidemia, including high cholesterol and triglyceride levels, which affected 14.8% of the residents (*n* = 17). Many residents reported undergoing annual health checkups (Fig. 2D).

**Fig. 3:**
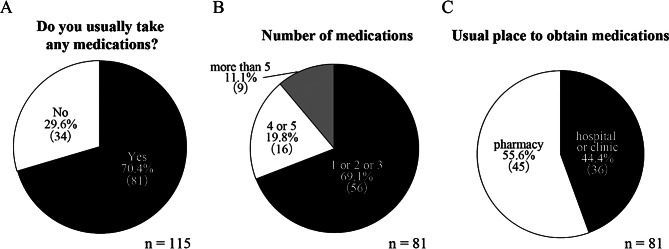
Medication use patterns among Toyone Village residents. (**A**) Prevalence of regular medication use among survey respondents (*n* = 115). (**B**) Number of different medications taken by respondents who reported using medications (*n* = 81). (**C**) Primary locations where respondents obtain their medications (*n* = 81)

Regarding medication use, 70.4% (*n* = 81) of the residents reported taking medications (Fig. 3A), with the majority taking one to three different types (Fig. 3B). The places from which residents usually obtained medicines were almost equally divided between pharmacies and hospitals/clinics (Fig. 3C).

### Dental health status

**Fig. 4:**
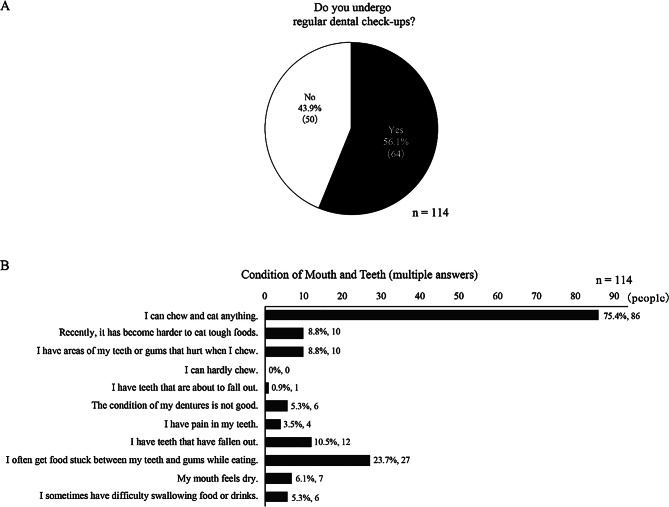
Dental health status among Toyone Village residents (*n* = 114). (**A**) Frequency of regular dental check-up attendance among survey respondents (*n* = 114). (**B**) Self-reported oral health conditions and chewing abilities (multiple answers allowed, *n* = 114)

Only 56.1% (n = 64) of the participants had regular dental checkups at least once a year (Fig. 4A). In terms of dental health, 75.4% (n = 86) of the participants reported being able to chew and eat everything (Fig. 4B). Of the 114 respondents who answered the dental health status, 24.6% (n = 28) were classified into the “decreased chewing ability group.” Moreover, 60.7% (n = 17) of this group were aged 65 years or older compared to the “maintained chewing ability group” (p < 0.05). Only 46.4% (n = 13) in the decreased chewing ability group reported having regular dental checkups, a lower proportion than that of the overall population. Furthermore, only 39.3% (n = 11) of the participants in the decreased chewing ability group reported being in good health, which was significantly lower than that of the maintained chewing ability group (p < 0.05).

### Nutrient intake and macronutrient balance

**Fig. 5:**
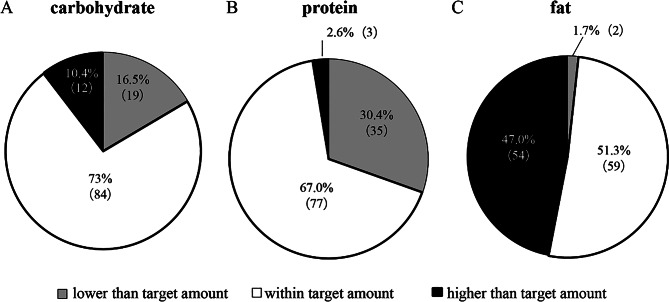
Macronutrient intake distribution relative to recommended targets (*n* = 115). (**A**) Carbohydrate intake distribution among respondents compared to target amounts (50–65% of total energy intake). (**B**) Protein intake distribution among respondents compared to target amounts (13–20% of total energy intake). (**C**) Fat intake distribution among respondents compared to target amounts (20–30% of total energy intake)

The mean distribution of energy from carbohydrates, proteins, and fats were 55.9%, 14.3%, and 29.7%, respectively. Only 25.2% (*n* = 29) of the respondents met the target intake for all three major nutrients. Many respondents tended to fall below the target protein intake, while a trend towards higher-than-recommended fat intake was observed (Fig. 5).

The mean daily vegetable intake was 197.7 g for males and 250.7 g for females (Table 2). When analyzed based on meal preparation involvement, individuals who prepared their own meals consumed an average of 238.3 g of vegetables per day, compared to 207.2 g among those who did not (*p* = 0.056).

**Table 2:** Vegetable and salt intake

	Male (*n* = 58)	Female (*n* = 57)
Vegetable (g)	197.7 (100.5)	250.7 (101.7)
Green and yellow vegetables (g)	78.9 (45.6)	94.0 (42.2)
Other vegetables (g)	118.8 (66.6)	156.7 (70.3)
Salt (g)	9.94 (3.58)	10.4 (3.27)

Parentheses show the standard deviation (SD)

The mean daily salt intake was 9.94 g for males and 10.4 g for females (Table 2). The mean salt intake of residents with hypertension (*n* = 33) was 11.3 g per day, which was significantly higher than that of residents without hypertension (9.7 g per day) (*p* < 0.05).

## Discussion

This study provides valuable insights into the significant challenges rural mountainous communities face regarding healthcare and food access and their potential impact on dietary intake and overall health, contributing to health inequities. Our findings from Toyone Village underscore the complex interplay between geographical isolation, dietary behaviors, and health outcomes, suggesting that leveraging accessible, community-based health resources may be a viable strategy to mitigate these inequities.

A significant proportion of participants had to travel over an hour one way to shop for food, with many having to stock up for more than one week at a time. This restricted access could impact both food procurement and dietary habits. Limited food access is associated with an unbalanced nutrient intake [7]. While this study did not include a direct comparison group from an urban area or a region with better food access, the reported travel times represent a considerable burden that may exacerbate existing disparities. The impact of limited food access was potentially reflected in vegetable consumption, with male residents consuming notably fewer vegetables (197.7 g per day) than the national average (262.2 g per day), while female residents’ intake (250.7 g per day) was comparable to the national level (250.6 g per day) [14]. This sex-based difference in vegetable consumption appeared to be closely related to meal preparation practices. Our analysis revealed that 89.3% of females were involved in preparing their own meals compared to only 37.9% of males. Furthermore, participants who were involved in meal preparation had a higher vegetable intake (238.3 g) than those who were not (207.2 g), although this difference was not statistically significant (*p* = 0.056). Although this result should be interpreted with caution, the observed trend, along with the pronounced sex gap in meal preparation, suggests that male residents’ lower vegetable intake is more likely due to their limited involvement in meal preparation rather than food access alone.

Regarding macronutrient distribution, the mean proportions of energy from carbohydrates, proteins, and fats (55.9%, 14.3%, and 29.7%, respectively) were comparable to the national averages (56.0%, 15.1%, and 28.9%, respectively) [14]. Thus, the overall pattern of lower protein and higher fat intake is not unique to Toyone Village but is consistent with broader trends observed at the national level. However, in this rural mountainous community, structural factors such as limited access to fresh food supplies and reliance on infrequent, large-volume shopping trips may further constrain the ability of residents to secure sufficient high-quality protein while increasing dependence on shelf-stable or processed foods that are often higher in fat. These contextual constraints may make it more difficult to correct macronutrient imbalances once they occur. Given the village’s markedly aged population, even a modest shortfall in protein intake and excess fat intake could have more serious consequences, potentially contributing to an increased risk of sarcopenia and frailty [15–17]. Although the prevalence of chronic diseases such as diabetes and dyslipidemia was not particularly high in the study population, the observed dietary habits suggest that residents may be at an increased risk for lifestyle-related diseases, potentially widening health disparities if unaddressed.

A particularly concerning finding was the relationship between salt intake and hypertension. The mean daily salt intake was 9.94 g for males and 10.4 g for females, showing an interesting deviation from national averages (males: 10.7 g, females: 9.1 g) [14]. Female residents in our study area had a higher salt intake than the national average, whereas male residents had a slightly lower intake. This unique pattern might reflect the regional dietary habits and food preservation practices common in rural areas. Residents with hypertension had a significantly higher salt intake (11.3 g per day) than that of those without hypertension, which considerably exceeded the recommended levels. This pattern is consistent with epidemiological studies of patients with hypertension [18, 19] and suggests that current disease management strategies may not adequately address dietary behaviors. These observations highlight the importance of personalized dietary advice tailored to specific health conditions, particularly in settings where regular access to healthcare professionals may be limited. Community pharmacies, as accessible points of contact, may be well positioned to provide this crucial support, integrating dietary counseling with medication management for chronic conditions.

Our study identified significant associations between dental health and overall health status. Patients with a reduced chewing ability were more likely to be older and less likely to undergo regular dental checkups. This group reported poor general health, highlighting the interconnected nature of oral health and overall health status. Overall, 56.1% of the participants reported having undergone dental checkups in the past year, comparable to the national average of 58.8% [14]. While this rate seems adequate, our findings emphasize the need for integrated strategies to improve oral health and nutritional support in rural mountainous areas, particularly for vulnerable groups experiencing limited access.

Given these challenges, innovative approaches to healthcare delivery and health promotion are required in depopulated rural mountainous areas to foster greater health equity. Our findings revealed several key challenges, including limited access to food stores affecting dietary habits, imbalanced macronutrient intake, and the need for professional support in both oral and nutritional management. The critical shortage of healthcare professionals in these areas necessitates alternative service delivery models. In Japan, national policy documents such as the “Pharmacy Vision for Patients” (2015) [20] explicitly position community pharmacies as community-based health-support hubs, with roles that include health promotion, lifestyle counseling, and nutritional support. These policy frameworks indicate that, in principle, community pharmacies are already expected to engage in nutrition-related health support activities and could serve as accessible contact points for residents. Consistent with this policy direction, a previous study reported that oral health check-ups conducted at community pharmacies were highly accepted by dentists and were expected to facilitate collaboration with dental clinics to maintain and improve residents’ oral health [21]. Although Toyone Village currently lacks a local pharmacy, our survey identified a crucial entry point: nearly 40% of the residents obtain medication from out-of-village pharmacies. This suggests that residents maintain regular contact with community pharmacy despite geographical barriers. Furthermore, 36.5% of participants expressed interest in receiving nutritional counseling, indicating a substantial unmet demand for accessible dietary support. Therefore, existing regular visits to neighboring community pharmacies can be leveraged for face-to-face dietary and oral health monitoring. Moreover, to further enhance the quality of care, establishing remote connections with urban pharmacies—where specialized staff such as registered dietitians are more concentrated—is a vital direction. Telepharmacy models [22] can bridge the geographical gap, enabling residents to access specialized nutritional support from remote professionals without physical travel.

However, these potential roles for community pharmacies should be regarded as hypotheses generated by our findings rather than definitive evidence of effectiveness. Future studies are needed to evaluate whether and how community pharmacy-based nutritional and oral health support can help reduce health disparities in depopulated rural areas.

Our study has several important limitations. First, there is a risk of response bias. Although the survey targeted all residents of the village, responses were received from only approximately 10% of the population, which may reflect participation by a particular subset of the community. Specifically, the proportion of participants aged 65 years and older in our study was 38.3%, which is lower than the actual aging rate of Toyone Village (52.4% according to the 2020 Census) [23], suggesting an under-representation of the elderly population. Second, the contextual characteristics of the study site limit the generalizability of our findings. This study was conducted in a single rural mountainous village. Toyone Village is a small, depopulated community with a markedly aging population exceeding 50%, described in Japan as a “marginal village,” where more than half of residents are aged 65 years or older and the community is at risk of functional decline. Local healthcare infrastructure is limited, relying on part-time internal medicine and dental clinics that operate only on designated days, and there is no community pharmacy in the village. Many residents must travel long distances to reach food stores and medical facilities, with restricted public transportation. Additionally, the survey was conducted during the winter, which may have influenced travel times and food procurement patterns in this mountainous setting. These features may not be shared by all rural or mountainous communities, and therefore our findings should be generalized to other settings with caution. Third, the study design and measurement methods impose further limitations. Because of the cross-sectional nature of the study, we cannot draw causal conclusions regarding the relationships among food access, dietary habits, and health outcomes. In addition, comparisons of absolute intake values with national averages, such as those from the National Health and Nutrition Survey (NHNS), should be interpreted with caution. Our study used the FFQg, whereas the NHNS employs a different dietary assessment methodology to estimate nutrient intake for the population. Such methodological differences can lead to variations in reported values and limit direct comparability of absolute intakes. Finally, our analyses were primarily descriptive. We did not employ multivariable analyses to systematically assess inter-variable relationships among food access, dietary intake, and health indicators, which limits the depth of our conclusions regarding these associations. Future studies with larger samples and more detailed analytical approaches will be needed to address these issues.

Future research should address these limitations and expand our understanding of healthcare access and dietary intake in rural mountainous areas. Longitudinal studies tracking changes in dietary habits and health outcomes over multiple seasons would provide valuable insights into temporal variations and causal relationships. Multi-region comparative studies across different rural mountainous areas, ideally including comparisons with urban or more accessible settings, would help establish the generalizability of our findings and identify region-specific challenges and solutions to address health disparities. Additionally, intervention studies evaluating the effectiveness of nutritional counseling programs delivered by community health professionals (e.g., dietitians, pharmacists) could provide evidence-based strategies for improving healthcare delivery in these areas. Such comprehensive research efforts would contribute to developing more effective, tailored interventions for maintaining and improving health outcomes in rural mountainous communities.

## Conclusions

This study revealed that residents in a rural mountainous community face significant challenges in healthcare and food access, which are linked to suboptimal nutrient intake. Key findings include the association between poor oral health and lower subjective well-being, and a high salt intake among hypertensive patients, highlighting specific areas for intervention. These results underscore the need for comprehensive health-promotion strategies. Such strategies should address not only access issues but also provide personalized nutritional and health support, potentially through the collaborative efforts of community-based health professionals, to effectively reduce health inequities in these vulnerable populations.

## Data Availability

Data supporting the findings of this study are available from the corresponding author upon reasonable request. The data are not publicly available because of privacy and ethical restrictions.

## Abbreviations

FFQg: Food frequency questionnaire based on food groups
NHNS: National Health and Nutrition Survey
